# Stathmin1 Plays Oncogenic Role and Is a Target of MicroRNA-223 in Gastric Cancer

**DOI:** 10.1371/journal.pone.0033919

**Published:** 2012-03-28

**Authors:** Wei Kang, Joanna H. M. Tong, Anthony W. H. Chan, Raymond W. M. Lung, Shuk Ling Chau, Queenie W. L. Wong, Nathalie Wong, Jun Yu, Alfred S. L. Cheng, Ka Fai To

**Affiliations:** 1 Department of Anatomical and Cellular Pathology, State Key Laboratory in Oncology in South China, Sir Y.K. Pao Center for Cancer, The Chinese University of Hong Kong, Hong Kong, China; 2 Institute of Digestive Disease, Li Ka Shing Institute of Health Sciences, Prince of Wales Hospital, The Chinese University of Hong Kong, Hong Kong, China; 3 Department of Medicine, The Chinese University of Hong Kong, Hong Kong, China; Vanderbilt University Medical Center, United States of America

## Abstract

Stathmin1 (STMN1) is a candidate oncoprotein and prognosis marker in several kinds of cancers. This study was aimed to analyze its expression and biological functions in gastric cancer. The expression of STMN1 was evaluated by qRT-PCR, western blot and immunohistochemistry. The biological function of STMN1 was determined by MTT proliferation assays, monolayer colony formation and cell invasion assays using small interference RNA technique in gastric cancer cell lines. We also explored the regulation of STMN1 expression by microRNA-223. STMN1 was upregulated in gastric cancer cell lines and primary gastric adenocarcinomas. STMN1-positive tumors were more likely to be found in old age group and associated with p53 nuclear expression. In diffuse type gastric adenocarcinomas, STMN1 expression was correlated with age (*p* = 0.043), T stage (*p* = 0.004) and lymph node metastasis (*p* = 0.046). Expression of STMN1 in diffuse type gastric adenocarcinoma was associated with poor disease specific survival by univariate analysis (*p* = 0.01). STMN1 knockdown in AGS and MKN7 cell lines suppressed proliferation (*p*<0.001), reduced monolayer colony formation (*p*<0.001), inhibited cell invasion and migration ability (*p*<0.001) and induced G1 phase arrest. siSTMN1 could also suppress cell growth *in vivo* (*p*<0. 01). We finally confirmed that STMN1 is a putative downstream target of miR-223 in gastric cancer. Our findings supported an oncogenic role of STMN1 in gastric cancer. STMN1 might serve as a prognostic marker and a potential therapeutic target for gastric cancer.

## Introduction

Gastric cancer is one of the most common malignancies and the second most frequent cause of cancer-related death worldwide. It has the highest incidence in China, Japan, Korea and eastern Asia. The overall prognosis is poor with a 5-year survival rate below 30% in most countries [1]. Several potential risk factors include high salt diet, smoking, low intake of fruits and vegetables, chronic gastritis with glandularatrophy and intestinal metaplasia, and *Helicobacter pylori (H. pylori)* infection. The clinical outcome of *H. pylori* infection has been shown to be influenced by various genetic factors, particularly *H. pylori*-virulence associated genes such as *cag*A, *vac*A, *ice*A and *bab*A [2]. *H. pylori* infection is also known to induce the expression of pro-inflammatory cyclooxygenase enzyme (COX-2) which shows upregulated expression in gastric cancer [3]. Previous studies have documented the importance of genetic and epigenetic alterations of oncogenes, tumor suppressor genes and mismatch repair genes in the development of gastric cancer. Protocadherin 10 [4], death-associated protein kinase [5], secreted frizzled-related protein [6] and peroxisome proliferator activated receptor gamma [7] have been shown to have reduced expression and tumor suppressor function in gastric carcinogenesis. On the other hand, retinoic acid-regulated nuclear matrix-associated protein [8] and yes-associated protein 1 [9] were both upregulated and exert oncogenic function in tumor development.

Stathmin1 (STMN1), also known as oncoprotein 18, is an important cytosolic microtubule-destabilizing protein which plays critical role in the process of mitosis through regulation of microtubule dynamics, and a variety of other biological processes [10]. High level of STMN1 expression is associated with poor prognosis in various malignancies including breast cancer [11], [12], prostate cancer [13], malignant mesothelioma [14], cervical cancer [15], and esophageal squamous cell carcinoma [16]. In 2010, Jeon et al. first reported that STMN1 over-expression was positively correlated with lymph node metastasis and advanced staging and vascular invasion, and negatively with recurrence-free survival in diffuse type gastric carcinoma [17]. The same group demonstrated the oncogenic role of STMN1 in gastric cancer by *in vitro* inhibition of proliferation, migration and invasion in gastric cell lines by knocking STMN1 down using siRNA, and *in vivo* inhibition of xenograft tumor growth in nude mice by siRNA transfection.

Regulation of STMN1 expression by miR-223 has been demonstrated in hepatocellular carcinoma by our previous study [18]. Micro-RNAs are a class of single-stranded RNA molecules of 21–23 base pair in length and regulate target genes expression through specific base-pairing interactions between miRNA and untranslated regions of targeted mRNAs [19]. MiRNAs would function as oncogenes or tumor suppressors in human cancers and are potentially used as novel diagnostic and prognostic biomarkers, and therapeutic targets. In gastric cancer, several miRNAs including miR-143 and -145 [20], miR-141 [21], miR-31 [22] and miR-106a [23] are downregulated, whereas some oncogenetic miRNAs such as miR-21 and miR-27a [24] are upregulated.

This study is aimed to investigate the functional role of STMN1 in gastric cancer development and mechanisms of regulation of STMN1 in gastric cancer.

## Results

### Up-regulation of STMN1 in gastric cancer cell lines and primary gastric cancer samples

**Figure 1:**
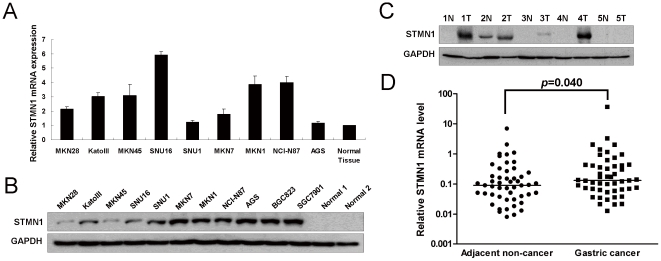
STMN1 upregulation in gastric cancer cell lines and primary gastric tumors. (A) STMN1 mRNA expression in gastric cancer cell lines compared with normal gastric mRNA commercially available from Ambion (AM7996). (B) STMN1 protein expression was assessed by Western blot in gastric cancer cell lines and normal gastric mucosa from patients underwent weight reduction gastric surgery. (C) Western blot of STMN1 in paired gastric cancer (T) and adjacent non-tumorous mucosal tissues (N). (D) STMN1 mRNA expression in 50 pairs of gastric adenocarcinoma and adjacent non-tumorous mucosa (*p* = 0.040).

The expression of STMN1 mRNA was higher in all 9 gastric cancer cell lines than the normal gastric tissue as shown in Fig. 1A. Western blot analysis confirmed the up-regulation of STMN1 protein in 11 gastric cancer cell lines (Fig. 1B). Up-regulated STMN1 protein expression was observed in 4 out of 5 primary gastric adenocarcinomas comparing with the corresponding non-tumorous gastric mucosa (Fig. 1C). QRT-PCR was conducted to investigate the STMN1 mRNA expression level. In primary gastric adenocarcinoma, 28 of 50 cases (56%) showed more than 1.5-fold up-regulation of STMN1 mRNA expression in tumor tissue compared with the corresponding non-tumorous mucosa. The mean level of STMN1 mRNA expression was significantly higher in tumor samples than that in the non-cancerous counterparts (*p* = 0.040, Fig. 1D).

### STMN1 expression correlates with poor prognosis in diffuse type gastric cancer

**Figure 2:**
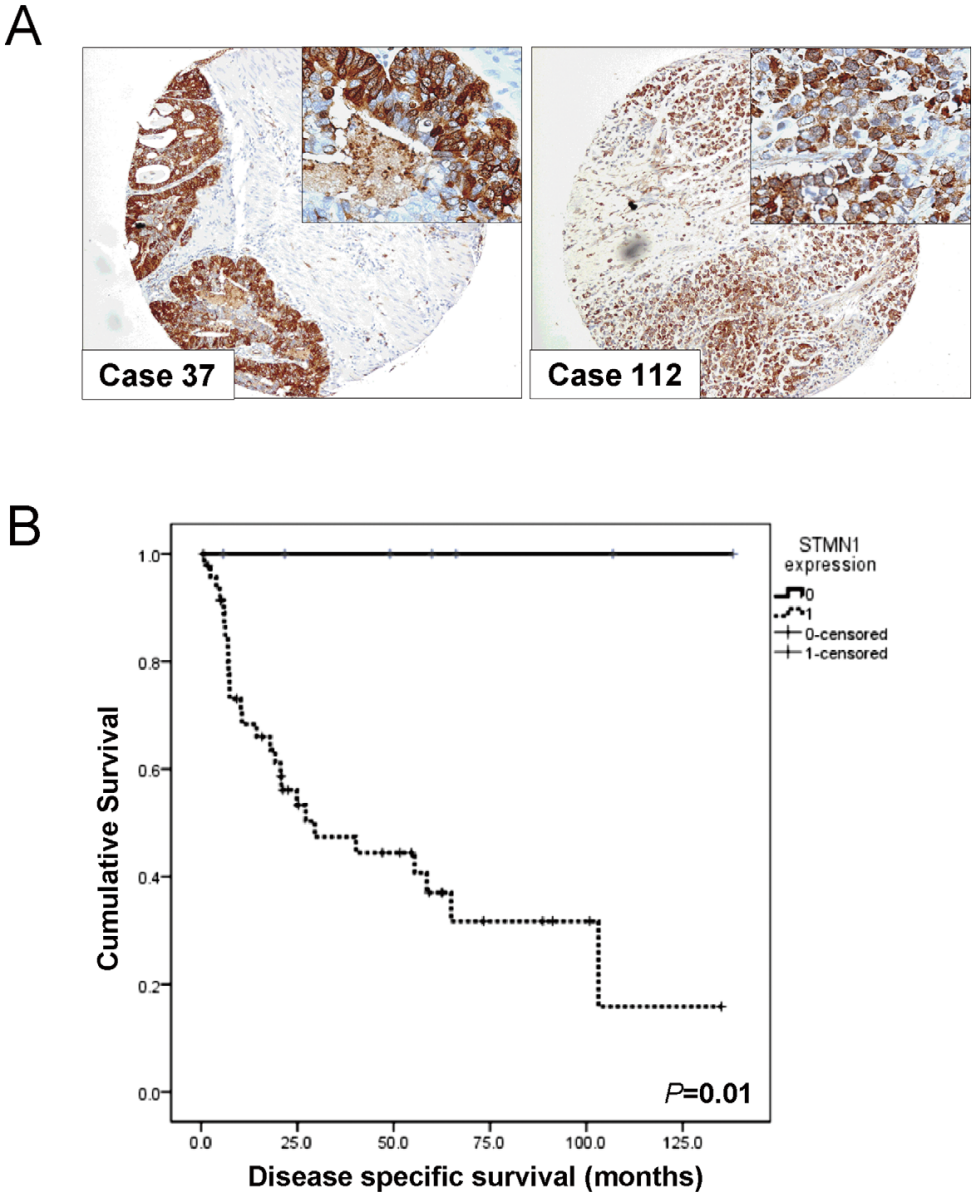
Clinical significance of STMN1 overexpression in gastric adenocarcinoma. (A) Representative photos of STMN1 immunohistochemistry in gastric cancer, case 37, intestinal type and case 112, diffuse type (original magnification ×100, insertion ×400). (B) Kaplan-Meier plot of disease-specific survival according to STMN1 expression status in diffuse type gastric adenocarcinoma.

Immunohistochemistry was performed to assess the STMN1 protein expression in 111 primary gastric adenocarcinoma samples. The STMN1 protein expression was mainly localized in the cytoplasm of the tumor cells (Fig. 2A). Positive immunoreactivity was observed in 96 gastric adenocarcinomas (86.5%). Among those STMN1-positive tumors, 40 showed strong (3+), 37 showed intermediate (2+) and 19 showed weak (1+) STMN1 staining. Previous study has demonstrated that the STMN1 expression was negatively regulated by tumor suppressor gene TP53 [25]. We therefore assessed the expression of p53 protein by immunohistochemistry and explored its correlation with STMN1 expression level. Aberrant nuclear p53 expression was found in 51 (45.9%) of gastric adenocarcinomas and more frequently in STMN1-positive tumor (50.0%) than STMN1-negative tumor (20.0%) (*p* = 0.03). The clinicopathologic characteristics of 111 patients with gastric adenocarcinoma and the association with STMN1 expression were shown in Table 1. STMN1-positive tumors were more likely to be found in old age group (*p* = 0.07) and associated with p53 nuclear expression (*p* = 0.03), Univariate analysis indicated that old age (*p*<0.036), histology with diffuse component (*p* = 0.012), stage (*p*<0.0001), T stage (*p* = 0.012), N stage (*p*<0.0001), M stage (*p*<0.0001) and the presence of lymph node metastasis (*p*<0.0001) correlated with poor disease-specific survival. By multivariate Cox proportional hazards regression analysis, only age (*p*<0.0001) and stage (*p*<0.0001) were independently associated with disease-specific survival (Table 2). In diffuse type gastric adenocarcinoma, STMN1 expression was associated with old age (*p* = 0.043), T stage (*p* = 0.004) and the presence of lymph node metastasis (*p* = 0.046). Expression of STMN1 in diffuse type gastric adenocarcinoma was associated with poorer disease-specific survival by univariate analysis (*p* = 0.01, Fig. 2B).

**Table 1:** Correlation of STMN1 expression with other clinicopathologic features.

		All cases (n = 111)			Diffuse type (n = 54)		
		STMN1 expression			STMN1 expression		
		Negative	Positive	*p*-Value	Negative	Positive	*p*-Value
Sex	M	10	67		4	34	
	F	5	29	NS	3	13	NS
Age	< = 60	10	40		6	21	
	>60	5	56	**0.07**	1	26	**0.043**
Type	Intestinal	8	48				
	Diffuse	7	47	NS			
Grade	1	0	2				
	2	7	32				
	3	8	61	NS	7	47	NA
Stage	I	4	29		3	6	
	II	1	9		0	2	
	III	3	30		0	20	
	IV	7	27	NS	4	19	NS
Stage (T)	1	4	19		3	2	
	2	3	32		1	17	
	3	6	43		3	28	
	4	2	1	NS	0	0	**0.004**
Stage (N)	0	4	26		3	6	
	1	6	22		2	8	
	2	2	29		0	20	
	3	3	18	NS	2	13	NS
Stage (M)	0	11	82		4	38	
	1	4	13	NS	3	9	NS
Lymph Node	0	4	26		3	6	
	1	11	69	NS	4	41	**0.046**
*H. pylori*	Absence	8	58		4	31	
	Presence	7	37	NS	3	16	NS
p53	0	12	48		5	22	
	1	3	48	**0.03**	2	25	NS

(NS, not significant; NA, not available).

**Table 2:** *P*-value of univariate and multivariate analysis of the association between clinicopathologic features and disease specific survival in patients with gastric adenocarcinoma.

	All cases (n = 111)		Diffuse type (n = 54)	
	Univariate	Multivariate	Univariate	Multivariate
Sex	0.077	NS	NS	NS
Age	**0.036**	**<0.0001**	**0.004**	**0.003**
Type	**0.012**	NS	NA	NA
Grade	0.356	NS	NA	NA
Stage	**<0.0001**	**<0.0001**	**0.039**	**0.008**
Stage (T)	**0.012**	NS	0.238	NS
Stage (N)	**<0.0001**	NS	0.052	NS
Stage (M)	**<0.0001**	NS	0.147	NS
*H. pylori*	0.124	NS	0.683	NS
Lymph Node	**<0.0001**	NS	**0.026**	NS
STMN1	0.091	NS	**0.01**	NS

(NS, not significant; NA, not available).

### Silencing of STMN1 inhibits the aggressive phenotype *in vitro*

**Figure 3:**
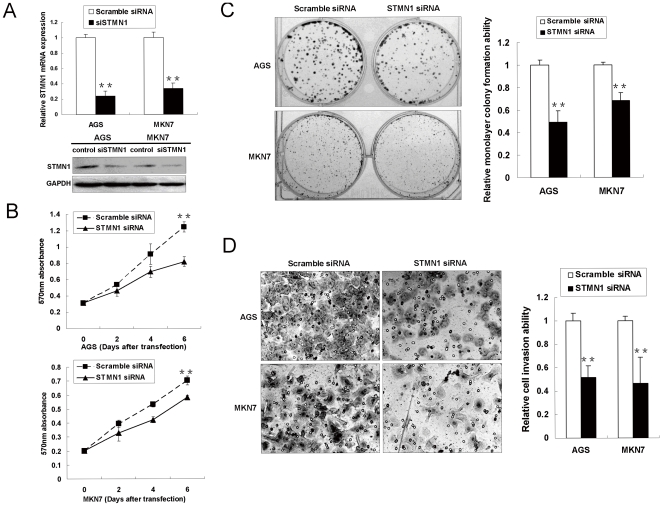
Knockdown of STMN1 by siRNA in gastric cancer cell lines AGS and MKN7. (A) Transfection of STMN1 siRNA successfully reduced STMN1 mRNA and protein expression in AGS and MKN7 cells (**, *p*<0.001). (B) MTT assays suggested knockdown STMN1 significantly suppressed proliferation in AGS and MKN7 (**, *p*<0.001). (C) Monolayer colony formation assays suggested transfection with siSTMN1 could reduce anchorage-dependent colony formation in AGS and MKN7 cell (**, *p*<0.001). All the experiments were performed in triplicate and the error bars represent standard deviations. (D) Representative images of cells invaded through the Matrigel-coated membrane to the underside of the micropores are shown. Significant reduction in the invasive ability was shown on STMN1 knockdown (**, *p*<0.001). The cell number was counted in 5 random view fields and the error bars represented standard deviations.

Frequent upregulation of STMN1 mRNA and protein in gastric tumors suggested a potential oncogenic role of this gene. Knockdown of STMN1 by small RNA interference (siRNA) markedly lowered mRNA and protein level (Fig. 3A) and significantly reduced cell proliferation in AGS and MKN7 cells as demonstrated by MTT assays (*p*<0.001, Fig. 3B). STMN1 siRNA-mediated growth suppressive effect was further confirmed by anchorage-dependent monolayer colony formation assay. A significant reduction of colony numbers was observed in cells transfected with STMN1 siRNA, compared with scramble controls in monolayer culture (reduced to 49.2% and 68.4% of scramble controls in AGS and MKN7 respectively; *p*<0.001, Fig. 3C).

In cell motility assays, a significant reduction in the invasive phenotype through the Matrigel-coated Boyden chamber (*p*<0.001, Fig. 3D) was demonstrated in STMN1 siRNA-transfected AGS and MKN7 cells (reduced to 51.6% and 46.8% of scramble controls in AGS and MKN7 respectively). In cell migration assays using Transwell Permeable Supports, a significant decrease in the number of cells migrating through the microporous membrane (*p*<0.001, Figure S1) was found in STMN1 siRNA transfected AGS and MKN7 cells (reduced to 65.9% and 42.7% of the scramble controls in AGS and MKN7, respectively), suggesting siSTMN1 could inhibit the migration ability of gastric cancer cells.

**Figure 4:**
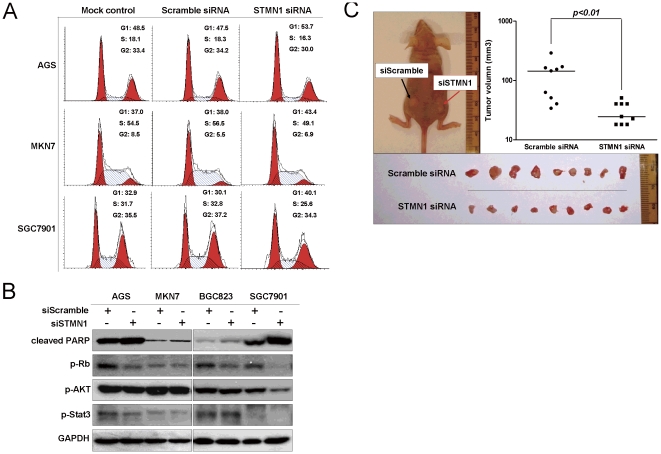
siSTMN1 induces G1 phase arrest in gastric cancer cells and inhibits cell growth *in vivo*. (A) Flow cytometric analysis revealed the accumulation of cells in G1 phase 24 hours after siSTMN1 treatment. Representative data from two independent experiments was shown. (B) Western blot analysis showed p-Rb (S807/811) reduction and increase of cleaved-PARP after STMN1 knockdown. p-AKT (S473) and p-Stat3 (T705) showed no difference. (C) siSTMN1-SGC7901 formed smaller xenograft tumors than siScramble-SGC7901 3 weeks after injection (*p*<0.01).

Since a growth inhibitory effect was observed in siSTMN1 transfected cells, we analyzed the transfectants for cell cycle parameters using flow cytometry. Twenty-four hours after transfection, accumulation of G1 cells was observed in siSTMN1 transfectants compared with the scramble siRNA controls (Fig. 4A). Cells in the G1 phase were increased from 47.5% to 53.7% in AGS, 38.0% to 43.4% in MKN7, and 30.1% to 40.1% in SGC7901 cells. And this was accompanied with a decrease of S phase cells. In keeping with the cell cycle arrest found in flow cytometric analysis, we observed a significant reduction of phospho-Rb (S807/811) in siSTMN1 transfectants (Fig. 4B). Furthermore, siSTMN1 could induce late apoptosis in four gastric cancer cell lines tested, AGS, MKN1, BGC823 and SGC7901, which was represented by an increase of cleaved-PARP (Fig. 4B). However, no significant difference was found in the level of p-AKT (S473) and p-Stat3 (T705) between siSTMN1 and negative control transfected.

### siSTMN1 inhibits the growth of gastric tumor *in vivo*

To further investigate the effect of STMN1 on *in vivo* growth of gastric tumor, siSTMN1 and scramble-transfected gastric cancer cells were injected subcutaneously to the right and left dorsal flank of nude mice, respectively. Since AGS and MKN7 cells do not form xenografts in nude mice, we used SGC7901 cells for *in vivo* study. siSTMN1-transfectant formed smaller tumors on the right dorsal flank than scramble controls on the left dorsal flank 3 weeks after injection (*p*<0.01, Fig. 4C).

### STMN1 is a downstream target of miR-223 in gastric cancer

**Figure 5:**
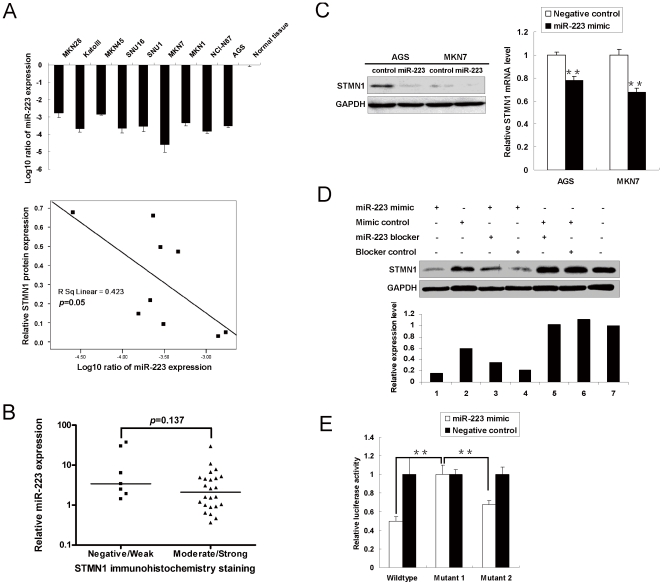
STMN1 is a putative downstream target of miR-223 in gastric cancer. (A) MiR-223 expression level in 9 gastric cancer cell lines as determined by qRT-PCR. A borderline correlation was observed between STMN1 protein level and reduced miR-223 expression (*p* = 0.05). (B) MiR-223 expression in 31 primary gastric adenocarcinomas stratified by STMN1 protein level. (C) MiR-223 down-regulated endogenous STMN1 mRNA and protein expression (**, *p*<0.001) in AGS and MKN7 cells. (D) Down-regulation of STMN1 protein expression by miR-223 was alleviated by miR-223 blocker in MKN7. (E) Luciferase reporter assays suggested STMN1 was a putative target of miR-223 (**, *p*<0.001). Wildtype: Luciferase construct containing wild type STMN1 3′UTR seed sequence; Mutant 1: the seed sequence was deleted; Mutant 2: 4-nucleotide mutations were introduced to the seed sequence.

As predicted by Targetscan, potential miR-223 binding site was found in the STMN1 3′UTR (position 12–18 of STMN1 3′UTR). Expression of miR-223 was downregulated in 9 gastric cancer cell lines compared with normal gastric epithelium tissue (Fig. 5A). Expression of miR-223 was negatively correlated with STMN1 protein expression (*p* = 0.05). We further assessed the STMN1 protein expression by immunohistochemistry and the level of miR-223 by qRT-PCR in 31 primary gastric cancer samples. Tumors with higher STMN1 immunoreactivity (score 2+ and 3+) showed a non-significant trend towards a lower miR-223 expression level (*p* = 0.137, Fig. 5B).

To demonstrate the potential suppressive effect of miR-223 on STMN1 expression, we transfected miR-223 to gastric cancer cell lines AGS and MKN7. MiR-223 suppressed STMN1 mRNA and protein expression in both cell lines (*p*<0.001, Fig. 5C). Adding miR-223 blocker rescued the STMN1 protein expression in MKN7 cells, suggesting the suppressive effect was specifically induced by miR-223 (Fig. 5D). Dual luciferase reporter assays were performed to study the interaction between miR-223 and STMN1 3′UTR (Fig. 5E). The reporter constructs containing predicted or mutated binding sites were co-transfected with miR-223 mimic to MKN28 cells, a gastric cancer cell line with relatively low endogenous STMN1 expression. MiR-223 exerted strong inhibitory effect on STMN1 3′UTR (49.7%, *p*<0.001). The inhibitory effect was eliminated when the seed region was deleted (Mutant 1), or alleviated when 4 nucleotides on the seed region were mutated (67.6%, *p*<0.001, Mutant 2).

## Discussion

In this study, we demonstrated the up-regulation of STMN1 expression in both mRNA and protein levels in gastric adenocarcinomas compared with normal gastric epithelium. The result suggested that STMN1 might have oncogenic function in gastric tumorigenesis. STMN1 has been reported to be a prognostic biomarker in several cancers including colorectal cancer [26], esophageal squamous cell carcinoma [16], hepatocellular carcinoma [27], [28], [29], [30] and oral squamous-cell carcinoma [31]. We demonstrated that STMN1 expression associated with old age group, advanced T stage, the presence of lymph node metastasis, and a shorter disease-specific survival time in diffuse type gastric adenocarcinoma. In keeping with this finding, Jeon et al. reported that STMN1 could predict poor prognosis in the diffuse type of gastric cancer and correlate with vascular invasion [17].

STMN1 regulates microtubule dynamics by promoting depolymerization of microtubules and preventing polymerization of tubulin heterodimers. Inhibition of STMN1 expression leads to accumulation of cells in the G2/M phases and is associated with severe mitotic spindle abnormalities and difficulty in the exit from mitosis [10]. STMN1 also mediates the effects of p27(Kip1) on cell motility [32]. In sarcoma cells [33] and non-small cell lung cancer [34], STMN1 stimulated cell motility in and through the extracellular matrix *in vitro* and increased the metastatic potential *in vivo*. In poorly differentiated gastric cancer cell lines SNU638 and SNU16, siRNA-induced STMN1 repression could suppress cell proliferation *in vitro* and *in vivo* [17]. In this study, we showed that siRNA knockdown of STMN1 inhibited cell proliferation and anchorage-dependent colony formation, impaired invasion and migration ability, induced G1 arrest and late apoptosis in gastric cancer cell lines. We further demonstrated that siSTMN1 inhibited *in vivo* growth of gastric cancer cell line SGC7901. Functional inhibition of STMN1 readily decreased cell proliferation and invasive phenotype, suggesting a protumorigenic role of STMN1 in gastric cancer.

MiR-223 is an evolutionarily conserved miRNA which was initially reported in granulopoiesis and myeloid differentiation [35]. The expression of miR-233 might be driven by the myeloid transcription factors, PU.1 and C/EBPs [36]. It could regulate several target genes such as Mef2c [37], a transcriptive factor that promotes myeloid progenitor proliferation. It also plays an essential role during osteoclast differentiation [38], and could be served as a potential biomarker for recurrent ovarian cancer [39] and sepsis [40]. We reported previously that STMN1 is a putative downstream target of miR-223 in hepatocellular carcinoma [18]. In this study, we observed a low miR-223 expression level in gastric cancer cell lines and an inverse relationship between miR-223 and STMN1 protein expression. By luciferase reporter assays, we confirmed the specific interaction between miR-223 and STMN1 3′UTR in gastric cancer cells. The negative modulation effect by miR-223 was further substantiated by a significantly reduced STMN1 protein level in gastric cancer cell lines after miR-223 re-expression. The results supported that STMN1 is a putative target of miR-223 in gastric cancer cells. Intriguingly, overexpression of miR-223 did not alter cell proliferation and apoptosis (Figure S2A & 2B) but significantly induced cell motility in gastric cancer cells (Figure S2C). In keeping with this finding, a recent study demonstrated that miR-223 promoted cell motility through post-transcriptional downregulation of tumor suppressor EPB41L3 in gastric cancer cells [41]. While a single miRNA can target multiple genes, multiple miRNA can regulate a single gene. The exact molecular mechanistic identification of how a given miRNA contributes to the phenotypic changes remains elusive.

Inactivation of tumor suppressor gene TP53 is the most common and most frequently studied molecular events in human cancer. It has been reported that p53 mediated the repression of STMN1 promoter activity, resulting in negative regulation of STMN1 expression and G2/M arrest in the cell cycle [25], [42]. It has been generally accepted that wild type p53 protein is not detectable by immunohistochemistry because it is unstable and has a relatively shorter half-life. Mutant p53 protein accumulated in the nucleus is relatively stable and has a longer half-life, which makes it detectable by immunohistochemistry. Therefore, a strong and diffuse immunoreactivity is generally indicative of mutant p53 [43]. We assessed the p53 status in gastric adenocarcinoma by immunohistochemistry and found that aberrant p53 immunoreactivity associated with higher STMN1 expression. It is therefore plausible that overexpression of STMN1 might in part due to inactivation of tumor suppressor gene p53 in gastric cancers.

In conclusion, we demonstrated the up-regulation of STMN1 in gastric adenocarcinoma and the expression was correlated with poor disease-specific survival in diffuse type gastric cancer. The oncogenic property of STMN1 in gastric tumorigenesis was confirmed by functional studies. We further demonstrated that the expression of STMN1 was negatively regulated by miR-223 in gastric cancer cells. Our finding suggested that STMN1 might serve as a prognostic marker and a potential therapeutic target for diffuse type gastric adenocarcinoma.

## Materials and Methods

### Cell line and cell culture

Eleven gastric cancer cell lines, MKN28, KATO-III, MKN45, SNU16, SNU1, MKN7, MKN1, NCI-N87, AGS, BGC823, SGC7901, were obtained from either the American Type Culture Collection (Rockville, MD, USA), RIKEN Cell Bank (Tsukuba, Japan) or as a gift from Institute Digestive Disease (IDD) of Prince Wales Hospital. These cell lines are grown in RPMI 1640 (GIBCO) supplemented with 10% fetal bovine serum (FBS, EU GIBCO), 100 U/ml penicillin and 10 µg/ml streptomycin in a humidified atmosphere of 5% CO_2_ at 37°C.

### Clinical gastric adenocarcinoma samples

A total of 111 gastric adenocarcinoma samples were retrieved from the tissue bank of Anatomical and Cellular Pathology, Prince of Wales Hospital, Shatin, Hong Kong. Another 5 pairs of primary tumors and adjacent non-tumorous tissues were collected during surgery from patients without any neoadjuvant therapy. The specimens were frozen immediately in −80°C for further molecular analysis. Biopsy specimens from 50 pairs of gastric cancer and the corresponding non-cancerous mucosa were kindly provided by IDD of Prince Wales Hospital. The study is approved by Joint Chinese University of Hong Kong–New Territories East Cluster Clinical Research Ethics Committee, Hong Kong (CREC Ref. No. 2009.521) and all participants provided written informed consent for the collection of samples and subsequent analysis.

### RNA extraction, qRT-PCR and microRNA qRT-PCR

Total RNA extraction was performed using Trizol reagent (Invitrogen) according to manufacturer's instruction. RNA concentration was measured by NanoDrop 1000 (Thermo Fisher Scientific). High-Capacity cDNA Reverse Transcription Kits (Applied Biosystems) were used for cDNA synthesis. For quantitative RT-PCR (qRT-PCR), Taqman Universal PCR Master Mix (Applied Biosystems) was applied for STMN1 (Sense: GAGGTCACGTGCCTCTGTTTG; Antisense: CTGACCACACTCTGAGCACCAA; Probe: Applied Biosystems, 185528556-1, FAM-CGCTTTTGTGCGCGC). The relative expression level was normalized with glyceraldehyde-3-phosphate dehydrogenase (GAPDH) and calculated using the 2∧ (-Delta Delta Ct) method. Taqman miRNA assays (Applied Biosystems) were used to quantify the expression levels of mature miR-223. Total RNA was reversed transcribed by MultiScribe (Applied Biosystems) in reaction mixture containing miR-specific stem-loop reverse transcriptive primer. All the reactions were performed in triplicates and water blanks were included as negative controls.

### Western blot and immunohistochemistry

Protein was extracted from gastric cancer cell lines and paired primary tissues using RIPA lysis buffer with proteinase inhibitor. Protein concentration was measured by the method of Bradford (Bod-Rad) and 20 µg of protein mixed with 2×SDS loading buffer was loaded per lane, separated by 12% SDS-polyacrylamide gel electrophoresis. STMN1 protein was detected with a polyclonal anti-STMN1 antibody (Cell Signaling, #3352, 1∶1000). Other antibodies are from Cell Signaling commercially, cleaved PARP (Asp214) (#9541, 1∶1000), phospho-Rb (Ser807/811) (#9308, 1∶1000), phospho-AKT (S473) (#9271, 1∶1000) and phospho-Stat3 (T705) (#9145, 1∶2000).

Immunohistochemistry was performed in 4 µm-thick sections from formalin-fixed and paraffin-embedded specimens. After de-waxing in xylene and graded ethanol, sections were incubated in 3% H_2_O_2_ solution for 25 minutes to block endogenous peroxidase activities and subsequently underwent microwaving in citrate buffer for antigen retrieval. The primary antibodies (1∶25 for STMN1 from Cell Signaling and 1∶100 for p53 from DAKO) were incubated at 4°C overnight and chromagen development was performed using the EnVision system (DAKO). The cytoplasmic expression of STMN1 was assessed by assigning a proportion score and an intensity score. The proportion score was according to proportion of tumor cells with positive cytoplasmic staining (0, none; 1, < = 10%; 2, 10 to < = 25%; 3, >25 to 50%; 4, >50%). The intensity score was assigned for the average intensity of positive tumor cells (0, none; 1, weak; 2, intermediate; 3, strong). The cytoplasmic score of STMN1 was the product of proportion and intensity scores, ranging from 0 to 12. The cytoplasmic expression was categorized into negative (score 0), 1+ (score 1 to 3), 2+ (score 4–6), and 3+ (score 7–12). Nuclear p53 expression in >10% of tumor cells was scored as aberrant overexpression.

### STMN1 functional assays

Transfection of STMN1 siRNA and scramble control (QIAGEN) was performed using Lipofectamine 2000 Transfection Reagent (Invitrogen). Cell proliferation was assessed using CellTiter 96 Non-Radioactive Cell Proliferation Assay (Promega) according to manufacturer's instruction. For colony formation assays in monolayer cultures, cells transfected with STMN1 siRNA or scramble control were cultured for 10 days. Cells were fixed with 70% ethanol for 15 minutes and stained with 2% crystal violet. Colonies with more than 50 cells per colony were counted. The experiments were repeated in triplicate.

The cell invasion assays were performed using BD Biocoat Matrigel Invasion Chambers (BD Biosciences). Transfected cells were seeded on the top chamber in culture medium containing 1% FBS, with complete medium (containing 10% FBS) added to the bottom chamber. After 24 hours incubation at 37°C, cells that invaded through the matrigel membrane were fixed with 100% methanol for 2 minutes and stained with 1% Toluidine blue for another 2 minutes. For statistics analysis, cells on the underside of the membrane were counted from 5 random microscopic fields (original magnification ×400). Each experiment was carried out in triplicate, and the mean value was expressed from 2 independent experiments.

The cell migration assays were performed using Transwell Permeable Supports (Corning, NY). Cells were harvested from culture dishes 24 hours after transfection, washed three times with culture medium and resuspended. Then 300 ul of the cell suspension (5×10^4^ cells) was added into the transwells, with 500 ul of culture medium containing 10% FBS in the lower chamber. After 24 hours incubation at 37°C, cells that could migrate through the microporous membrane were fixed with 100% methanol for 2 minutes and stained with 1% Toluidine blue for another 2 minutes. For statistics analysis, we counted the attached cell number in 3 view fields of each chamber randomly and took the average cell number of each field.

### Flow cytometry analysis for cell cycle arrest

For cell cycle analysis, AGS, MKN7 and SGC7901 cells were collecting at the time 24 hours after transfection in 6 cm plates. Before transfection, the cells underwent starvation for 12 hours for synchronization. Cells were harvested using cold PBS and fixed in 70% cold ethanol for overnight in 4°C and treated with 1 ng/ml RNase A for 10 minutes at 37°C. Cellular DNA was stained with 15 ng/ml propidium iodide (PI) for 30 minutes at 37°C in the dark. The cells then were sorted by FACS Calibur Flow Cytometer (Becton Dickinson, CA) and cell-cycle profiles were determined using the ModFitLT software (Becton Dickinson, San Diego, CA). The experiments were repeated for two separate times.

### *In vivo* tumorigenicity study

SGC7901 cells (2×10^6^cells suspended in 0.1 ml PBS) transiently transfected with siScramble and siSTMN1 were injected subcutaneously into the dorsal flank of nine 4-week-old male Balb/c nude mice (siSTMN1 on the right side and the negative control cells on the left). The xenografts were taken out and tumor diameter was measured and documented at the end of week 3. Tumor volume (mm^3^) was estimated by measuring the longest and shortest diameter of the tumor and calculating as follows: volume = (shortest diameter)^2^×(longest diameter)×0.5. The animal handling and all experimental procedures were approved by the Animal Ethics Committee of the Chinese University of Hong Kong.

### Luciferase reporter assays and miR-223 transfection

The putative miR-223 binding site at the 3′ untranslated region (3′UTR) of STMN1 was cloned into pMIR-REPORT vector (Ambion Inc.). Two mutant constructs were generated by either deletion or mutations of the complementary seed sequence to the miR-223 binding region, as described previously [18]. The firefly luciferase construct was co-transfected with Renilla luciferase vector control into MKN28 cells in the presence of either synthetic miR-223 molecules or scramble miRNA control. Dual luciferase reporter assays (Promega) were performed 36 hours after transfection. Lipofectamine 2000 was used for the transfection of miR-223 into AGS and MKN7 cells.

### Statistical analysis

The Student T test was used to compare the difference in biological behavior between STMN1 knockdown cells and scramble siRNA-transfected cells, and between miR-223-transfected and scramble miRNA transfected cells. Correlation between STMN1 expression and clinicopathologic parameters were assessed by nonparametric Spearman's rho rank test. The Kaplan-Meier method was used to estimate the survival rates for each variable. The equivalences of the survival curves were tested by log-rank statistics. For those variables being statistically significant found in the univariate survival analysis (*p*<0.05), the Cox proportional hazards model with the likelihood ratio statistics was employed to further evaluate them for multivariate survival analysis. All statistical analysis was performed by SPSS software (version 16.0; SPSS Inc). A two-tailed *p*-value of less than 0.05 was considered statistically significant and the *p*-value less than 0.001 was considered highly significant.

## Supporting Information

Figure S1 **siSTMN1 inhibits cell migration in gastric cancer.** Representative pictures of AGS and MKN7 cells, which were transfected with siSTMN1 and siScramble then migrated through a microporous membrane (**, *p*<0.001).
(TIF)

Figure S2 **The functional study of miR-223 in gastric cancer cell lines.** (A) MTT proliferative assays of AGS, MKN7 and SGC7901 after miR-223 transfection (4 days after transfection). (B) Western blot analysis of cleaved-PARP in AGS and MKN7 cells on 24 hours after miR-223 transfection. (C) Representative Matrigel invasion images of AGS, MKN7 and SGC7901 are shown. miR-223 enhanced the cell invasion ability of gastric cancer cells (*, *p*<0.01; **, *p*<0.001). The cell number was counted in 3 random view fields and the error bars represented standard deviations.
(TIF)
