# Supplementary figures and images for: Stathmin1 Plays Oncogenic Role and Is a Target of MicroRNA-223 in Gastric Cancer
Source: PLoS One. 2012 Mar 28;7(3):e33919. doi: 10.1371/journal.pone.0033919 (PMC3314670; doi:10.1371/journal.pone.0033919)

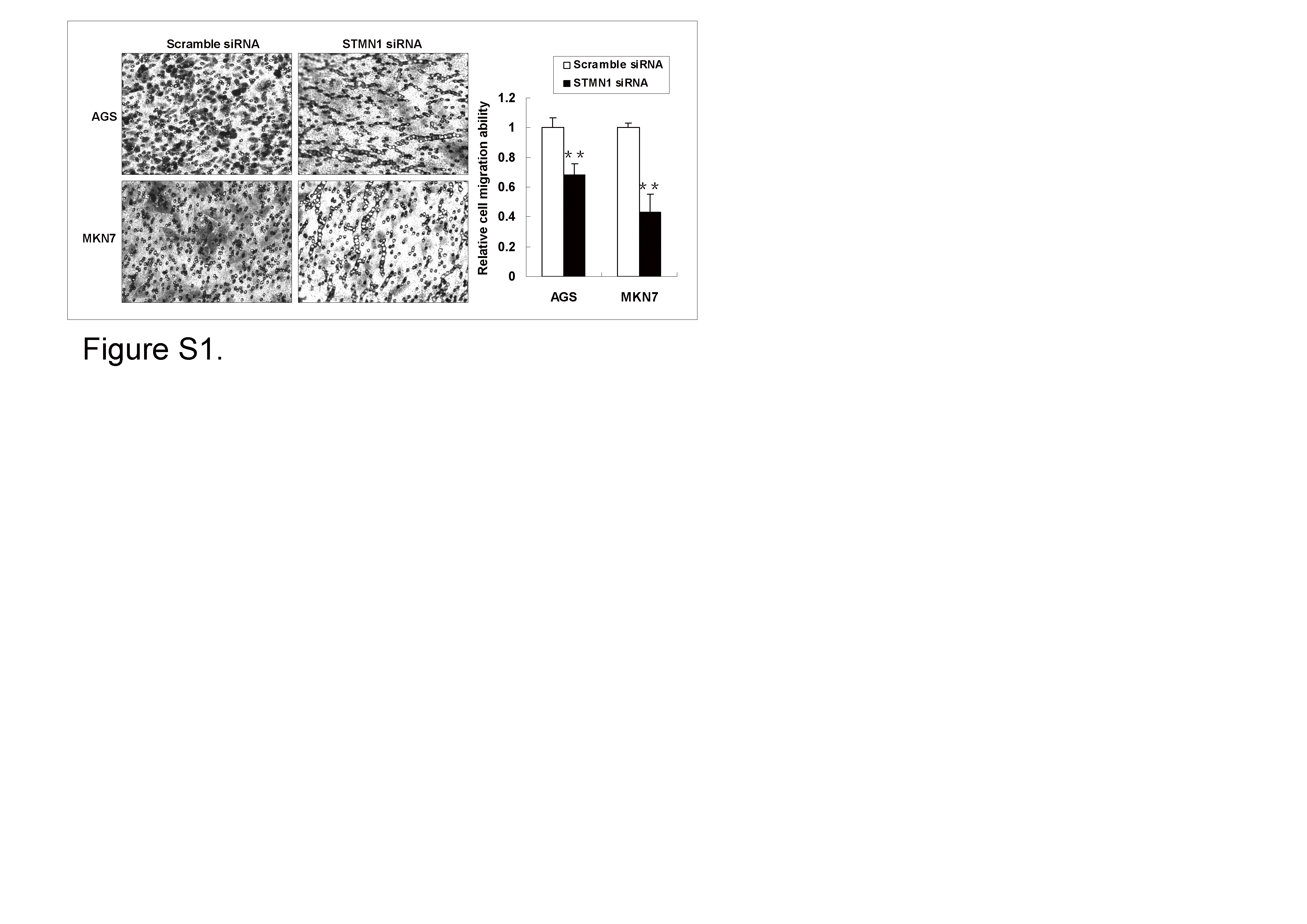

Supplement: Figure S1 — siSTMN1 inhibits cell migration in gastric cancer. Representative pictures of AGS and MKN7 cells, which were transfected with siSTMN1 and siScramble then migrated through a microporous membrane (**, p<0.001). (TIF) [file pone.0033919.s001.tif]

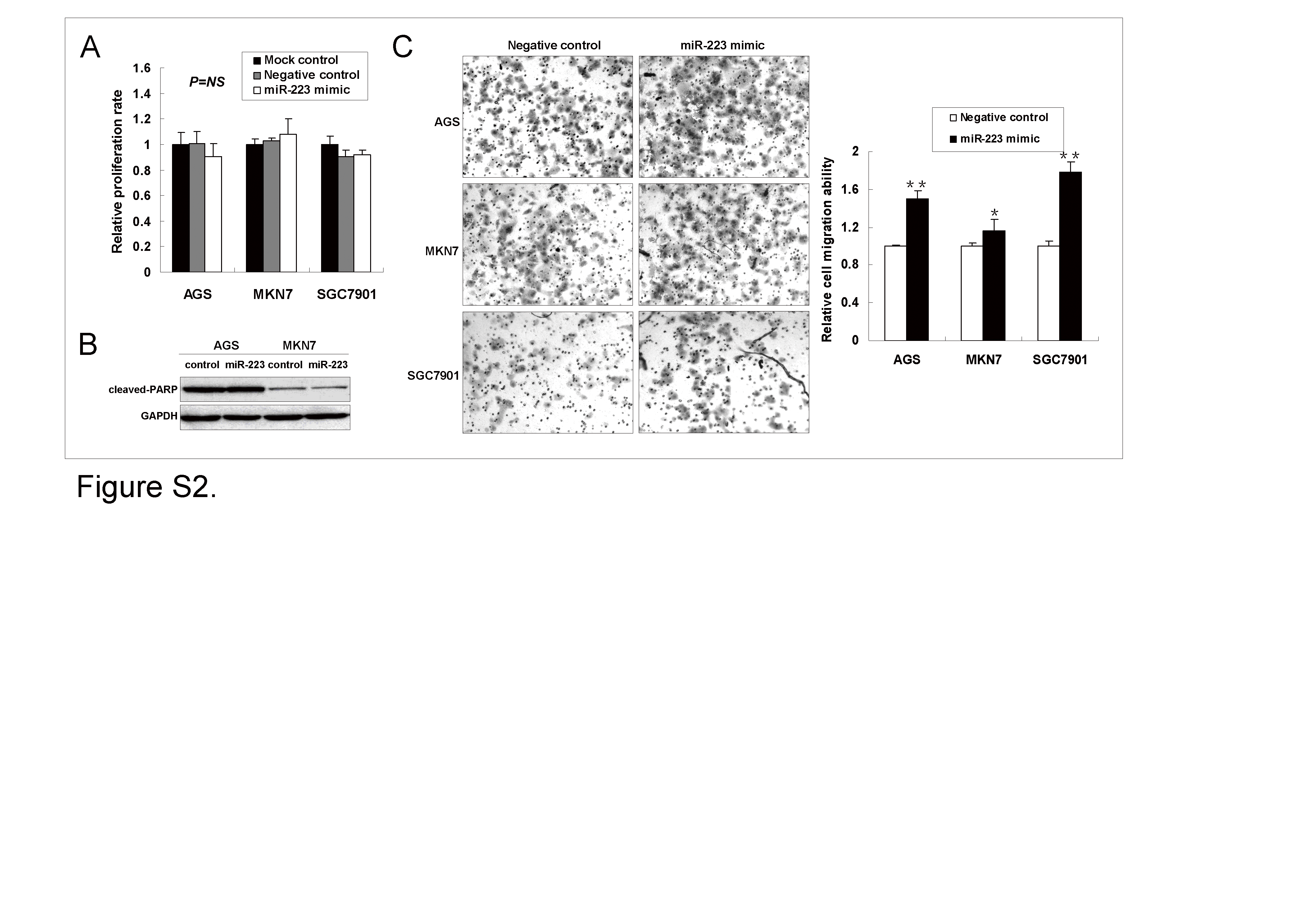

Supplement: Figure S2 — The functional study of miR-223 in gastric cancer cell lines. (A) MTT proliferative assays of AGS, MKN7 and SGC7901 after miR-223 transfection (4 days after transfection). (B) Western blot analysis of cleaved-PARP in AGS and MKN7 cells on 24 hours after miR-223 transfection. (C) Representative Matrigel invasion images of AGS, MKN7 and SGC7901 are shown. miR-223 enhanced the cell invasion ability of gastric cancer cells (*, p<0.01; **, p<0.001). The cell number was counted in 3 random view fields and the error bars represented standard deviations. (TIF) [file pone.0033919.s002.tif]
